# Intimate partner violence and women living with episodic disabilities: a scoping review protocol

**DOI:** 10.1186/s13643-022-01972-x

**Published:** 2022-05-18

**Authors:** Karen A. Campbell, Marilyn Ford-Gilboe, Meagan Stanley, Karen MacKinnon

**Affiliations:** 1 Arthur Labatt Family School of Nursing, Faculty of Health Science, The University of Western Ontario, 1151 Richmond St, London, Ontario N6A 3K7 Canada; 2 Allyn & Betty Taylor Library, The University of Western Ontario, 1151 Richmond St, London, Ontario N6A 3K7 Canada; 3 School of Nursing, University of Victoria, PO Box 1700 STN CSC, Victoria, British Columbia V8P 2Y2 Canada

**Keywords:** Episodic disability, Intimate partner violence, Lupus, Multiple sclerosis, Scoping review

## Abstract

**Background:**

Violence towards women with disabilities is most commonly perpetrated by current or former intimate partners and more than half of disabled women experience intimate partner violence in their lifetime. Disabilities differ by presence, type, and complexity, yet are commonly researched collectively. A more nuanced understanding of the relationship between intimate partner violence and episodic disability is required to better support women living with these concurrent challenges. The objective of this scoping review is to investigate and synthesize the literature reporting on intimate partner violence for women living with an episodic disability to identify key concepts and knowledge gaps on this topic. Ultimately, this review aims to improve health services for this stigmatized group of women with episodic disabilities.

**Methods:**

This scoping review will consider all studies that focus on women (18 years of age or older) who have experienced intimate partner violence and have an episodic disability. Episodic disabilities will include multiple sclerosis, chronic fatigue syndrome, fibromyalgia, lupus, or rheumatoid arthritis. The broad review question is what is known about intimate partner violence within the context of women living with an episodic disability? Databases to be searched include MEDLINE (OVID), CINAHL, Embase, PsychInfo, and Scopus with no limits on language or time frame. Joanna Briggs Institute methodology will guide this scoping review to address the review questions outlined in the protocol. For papers that meet the inclusion criteria, data will be extracted, and findings will be presented in tables and narrative form. A PRISMA table will be included to enhance the transparency of the process. A descriptive qualitative approach to analysis will be conducted following Braun and Clarke’s reflexive thematic analysis. The findings of the scoping review will be presented through a thematic narrative.

**Discussion:**

Findings from this review will be used to identify important priorities for future research based on knowledge gaps and inform both health care practices and health and social interventions for women living with intimate partner violence and episodic disabilities.

## Background

Intimate partner violence (IPV) is a pervasive, global public health issue impacting women across all settings, socioeconomic statuses, faith traditions and cultures [1]. It is defined as a pattern of behaviour by a current or former intimate partner that causes physical, sexual or psychological harm, including acts of physical aggression, sexual coercion, psychological abuse, and controlling behaviours [1]. Violence towards women with disabilities is most commonly perpetrated by current or former intimate partners and estimates suggest that 54% of disabled women experience IPV in their lifetime [2, 3]. Disabilities differ by presence, type, and complexity, yet are commonly researched collectively.

Episodic disabilities differ from permanent or progressive disabilities because they do not follow the typical illness trajectory of warning signs, illness, recuperation, and full recovery [4]. They are chronic conditions or diseases that are unpredictable in course and fluctuate between periods and levels of functional disability and wellness. Many episodic disabilities are autoimmune diseases, such as lupus or multiple sclerosis, and the risk of these conditions is gendered, with women being more susceptible than men [5]. Furthermore, the average age of onset for these conditions is between 20 and 40 years of age [6], which is also the most common age range for police-reported incidents of IPV [7].

Some aspects of episodic disabilities may further enhance women’s risk of IPV. These types of disabilities are often referred to as invisible illnesses because episodes of exacerbation may not be outwardly noticeable [6]. It is not uncommon for women with invisible disabilities to experience shaming and microaggressions as a result of their disability [8, 9]. The unusual and unobvious presentation of disability can decrease levels of support from others and negatively impact employment and overall quality of life [4, 10]. These experiences may lead to increased emotional and financial dependence on an intimate partner.

Many health-related symptoms of episodic disabilities are also frequently reported by women who have experienced IPV. Women presenting with fatigue, pain, paraesthesia, depression and anxiety, cognitive difficulties, and disordered sleeping could be exhibiting indicators of IPV or/and their episodic condition [6, 11]. Alvarez et al. [12] found that IPV was most often disclosed during assessments for anxiety and sleep problems. This poses a potential for healthcare practitioners to inadvertently overlook risk indicators of IPV, assuming symptoms are related to the episodic disability.

Because supportive care associated with IPV most often occurs in primary care settings, nurses, physicians, and other allied health professionals are in prime positions to assess for and respond to disclosures of violence [13]. Still, IPV remains underrecognized by healthcare professionals [14]. In one study, healthcare providers in outpatient care settings recognized the adverse effects of IPV and their role in screening for it; however, over two-thirds of providers surveyed indicated a lack of protocols for responding to IPV [15]. For many healthcare providers, including nurses, physicians, pharmacists, and paramedics, a lack of training and knowledge about IPV is a significant barrier to providing effective care to patients [13, 16, 17]. For women who are living with episodic disabilities, who often have a network of providers, many opportunities for supportive care could be overlooked.

Healthcare providers also face additional barriers, including that women underreport experiences of IPV [14]. Vranda and colleagues found that the majority of women in their study concealed experiences of IPV from mental health professionals because screening often occurred while their violent partner was present [18]. Given the high rate of anxiety and depression occurring in women with episodic diseases [19], these health professionals could provide essential services to improve health outcomes. To do this effectively, appropriate referrals and responses that extend beyond providing pamphlets or lists of available resources are needed [12, 20, 21].

A preliminary search of CINAHL, MEDLINE, the Cochrane Database of Systematic Reviews and JBI Evidence Synthesis was conducted and no current or underway systematic reviews or scoping reviews on the topic of IPV and episodic disabilities were identified. When searched for individual episodic disabilities in women and IPV, a variety of reviews were identified that focused on women with HIV [22]. Given the thorough examination of HIV in women and IPV, we will not include HIV as an episodic disability of interest in this scoping review. Also, because there is an array of episodic disabilities, we chose to focus on those that are disproportionately experienced by women.

The objective of this scoping review is to investigate and synthesize the literature reporting on IPV among women living with an episodic disability to identify key concepts and knowledge gaps on this topic. Findings from this review will be used to identify important priorities for future research based on knowledge gaps and inform both health care practices and health and social interventions for women living with IPV and episodic disabilities.

## Methods

The proposed scoping review will be conducted in accordance with the Joanna Briggs Institute (JBI) methodology for scoping reviews [23].

### Review question

This scoping review poses the broad review question: what is known about IPV within the context of women living with an episodic disability?

### Inclusion criteria

#### Participants

For this review, women are defined as any person self-identifying as a woman and who is 18 years of age or older. Papers and research reports that focus on or discuss separately women who have a diagnosis of one or more of the following episodic disabilities: multiple sclerosis, chronic fatigue syndrome, fibromyalgia, lupus, or rheumatoid arthritis will be included. Papers that focus on men, children and/or adolescents under the age of 18, or women living with HIV/AIDS will be excluded.

#### Concept

This review focuses on IPV which we defined as a pattern of behaviour by a current or former intimate partner that causes physical, sexual, or psychological harm, including acts of physical aggression, sexual coercion, psychological abuse, and controlling behaviours [1].

Papers that focus on women who are currently experiencing or have previously experienced one or more instances of IPV with a current or former partner will be included. Intimate partners can include marital or non-marital partners with a person of the same or a different gender.

Men experiencing IPV, perpetrators of IPV, and women experiencing domestic violence not by a current or former intimate partner (e.g., by a family member other than a partner) will be excluded.

#### Context

Sources of information from low-, middle-, and upper-income countries will be included in this review.

#### Types of sources

This scoping review will consider quantitative, mixed methods, and observational studies including prospective and retrospective cohort studies, case-control studies, and cross-sectional studies for inclusion.

Qualitative studies will also be considered that focus on qualitative data including, but not limited to, designs such as phenomenology, grounded theory, ethnography, qualitative description, action research, and feminist research. In addition, systematic reviews that meet the inclusion criteria will also be considered.

Grey literature, including reports, policy literature, working papers, government documents, and newsletters, will be included; conference abstracts and blogs will be excluded.

#### Search strategy

Articles will be selected using a three-step strategy as recommended by JBI [23]. The first step includes a limited search of two databases for relevant literature. Next, all sources will be analyzed for common text words found in titles and abstracts, and for index terms. Using these terms, a research librarian will conduct a comprehensive search of all databases using the terms identified during step two. The reference lists of identified sources will be searched for additional relevant texts. Unpublished sources will be searched using Google Scholar and ProQuest Dissertations and Theses.

The search strategy will aim to locate both published and unpublished studies. An initial limited search of CINAHL was undertaken to identify articles on the topic. The text words contained in the titles and abstracts of relevant articles, and the index terms used to describe the articles were used to develop a full search strategy for CINAHL (see Appendix 1). The search strategy, including all identified keywords and index terms, will be adapted for each included database and/or information source. The reference list of all included sources of evidence will be screened for additional studies.

Studies published in any language will be considered for inclusion. Although our search strategy will be limited to English search terms, papers written in other languages will be described in the review report as long as it is possible to obtain an English abstract to determine whether the paper meets our inclusion criteria. Tallying these papers will help to provide some idea of the international literature on this topic. There will be no limitations on the timeframe.

#### Information sources

The databases to be searched include MEDLINE (OVID), CINAHL, Embase, PsychInfo, and Scopus. Sources of unpublished studies and grey literature will extend to government sites and professional organizations or agencies that provide support for any of the included episodic illnesses.

#### Study/Source of evidence selection

Following the search, all identified sources will be uploaded into a citation management system (Endnote X9) and duplicates will be removed. Source selection will occur over a four-phase process based on a JBI framework for evidence selection [23]. First, a random sample of 25 titles and abstracts will be selected. Next, the first author (KC) and another reviewer will screen all 25 selections using the eligibility criteria and based upon the definitions in this scoping review protocol. The third phase includes a meeting of the reviewers to reconcile findings and discuss any discrepancies. Disagreements that occur during any phase of the selection process will be resolved through discussion and consensus decision making or with support from additional reviewers. Any decisions to refine the eligibility criteria based on these discussions will be made by consensus of all named authors. Finally, once a minimum agreement of 75% of selections is achieved, screening can commence. All decisions of the selection process (i.e., included and excluded sources, reasons for exclusion) will be presented in a Preferred Reporting Items for Systematic Reviews and Meta-analyses Extension for Scoping Reviews (PRISMA-ScR) flowchart [24].

#### Data extraction

Data will be extracted from all papers to be included in the scoping review by two independent reviewers. Extraction of the data be guided by the following questions: (a) how can we recognize IPV in this group and what do we know about assessment; (b) how can effective interventions be developed for this group of stigmatized women; and (c) how can these women be better supported by health care providers and social services? A draft data charting table, based on guidance from the JBI Manual for Evidence Synthesis [23], will guide the extraction process and will be refined and updated as needed during the review stage. JBI System for the Unified Management, Assessment and Review of Information (JBI SUMARI) will be used to organize the extracted data for the review report [25]. The initial step will include piloting the data charting table by having reviewers extract data from three sources to ensure all relevant details are obtained. Because this is a scoping review, sources of evidence will not be critically appraised [23].

#### Data analysis and presentation

The evidence presented will be descriptive in nature and respond directly to the review question: what is known about IPV within the context of women living with an episodic disability? A table will be created to capture basic information such as year of publication, country of origin, populations, sample sizes, concepts, and methodology/methods used. The health conditions cited will also be charted. PRISMA results will be captured in a flowchart. Any variances from this scoping review protocol will be noted and explained. A descriptive qualitative approach to analysis will be conducted following Braun and Clarke’s reflexive thematic analysis [26]. Consequently, the findings of the scoping review will be presented through a thematic narrative.

## Discussion

Findings from this review have the potential to inform how health and social service providers can support women with episodic disabilities who experience IPV. Emerging IPV interventions indicate increased effectiveness when approaches are tailored to women’s specific circumstances [21]. As clinicians and health researchers begin to understand the uniqueness of IPV experiences for women with differing disabilities, they will be able to better adapt their responses and interventions to women with specific disabilities. For women with episodic disabilities, this may be particularly important due to the shifting (or changing) nature of their conditions, the stigmatizing impacts of living with non-visible disabilities and the negative effects of stress on their health and disability progression [27–30]. This scoping review examines the population of women within the dual contexts of living with an episodic disability and experiencing IPV. Given that disability is typically researched collectively, it may be difficult to isolate the episodic disabilities of interest within this phenomenon. In addition, trauma, violence, and abuse in disability studies may report on more than just violence from intimate partners. For example, abusive care providers could be intimate partners or others. This may require a deep examination of sources to determine relevance. All reviewers will comprehensively assess during the selection phase in order to maximize relevant literature for review.

## Data Availability

Not applicable.

## Appendix

### Appendix 1: Search strategy

#### CINAHL

Search conducted in July 2021

**Table:**

Search	Query	Records retrieved
#1	(MH "Intimate Partner Violence") OR "intimate partner violence" OR “spousal abuse” OR "spousal violence" OR (MH "Dating Violence") OR "dating violence" OR (MH "Dating Violence") OR "dating violence" OR "intimate partner abuse"	17,349
#2	"episodic disabilities" OR (MH "Autoimmune Diseases") OR (MH "Arthritis, Rheumatoid") OR (MH "Lupus Erythematosus, Systemic+") OR (MH "Lupus Nephritis") OR Lupus OR arthritis OR (MH "Multiple Sclerosis") OR "multiple sclerosis" OR (MH "Fibromyalgia") OR "fibromyalgia" OR (MH "Fatigue Syndrome, Chronic") OR "chronic fatigue syndrome"	133,854
#3 (#1 AND #2)	(MH "Intimate Partner Violence") OR "intimate partner violence" OR “spousal abuse” OR "spousal violence" OR (MH "Dating Violence") OR "dating violence" OR (MH "Dating Violence") OR "dating violence" OR "intimate partner abuse" **AND** "episodic disabilities" OR (MH "Autoimmune Diseases") OR (MH "Arthritis, Rheumatoid") OR (MH "Lupus Erythematosus, Systemic+") OR (MH "Lupus Nephritis") OR Lupus OR arthritis OR (MH "Multiple Sclerosis") OR "multiple sclerosis" OR (MH "Fibromyalgia") OR "fibromyalgia" OR (MH "Fatigue Syndrome, Chronic") OR "chronic fatigue syndrome"	47

### Appendix 2: Data extraction instrument

**Table:**

Author(s)	
Year of publication	
Country of origin	
Aims	
Context/setting	
Study population (type of disability/condition) and sample size	
Concept(s) significant to the review	
Details of Violence	
Details of Intervention	
Details of Outcomes/Key Findings	

## Abbreviations

IPV: Intimate partner violence
JBI: Joanna Briggs Institute
